# Assessment of Quality of Life in Gestational Diabetes Mellitus Care—Study Protocol of the GDM-QOL Project

**DOI:** 10.3390/healthcare12010001

**Published:** 2023-12-19

**Authors:** Lisa Güldner, Klara Greffin, Holger Muehlan, Johannes Stubert

**Affiliations:** 1 University Gynecological Hospital and Polyclinic, University Medicine Rostock, 18059 Rostock, Germany; johannes.stubert@uni-rostock.de; 2 Department Health & Prevention, Institute of Psychology, University of Greifswald, 17489 Greifswald, Germany; klara.greffin@uni-greifswald.de (K.G.); holger.muehlan@uni-greifswald.de (H.M.)

**Keywords:** quality of life, gestational diabetes mellitus, pregnancy, maternal well-being, hyperglycemia, instrument development, patient-reported outcome

## Abstract

In recent years, the concept of quality of life (QoL) has gained significant importance within health care and clinical research, e.g., as in patient-reported outcomes. In gestational diabetes mellitus (GDM) care, enhancing QoL through reasonable interventions is considered equally important as achieving metabolic control and preventing complications in the treatment process, leading to the suggestion that QoL assessment should be implemented as a clinical standard in GDM care. Although a considerable number of questionnaires for the measurement of general as well as health-related and diabetes-specific QoL are frequently used in GDM research, a validated QoL questionnaire tailored to women with GDM does not exist in German-speaking countries. To develop and test such an instrument, we plan to conduct the following steps: (a) translate the Persian questionnaire GDMQ-36, the only GDM-specific questionnaire to date; (b) conduct expert ratings as well as pretests featuring cognitive debriefings and structured interviews with women suffering from GDM for evaluating comprehensibility, face and content validity; (c) pilot and validate the preliminary questionnaire in terms of testing its psychometric performance (e.g., via confirmatory factor analysis). The resulting GDM-specific questionnaire will facilitate a broader perspective of the pregnant women’s expectations, needs, impairments, and burdens related to their disease, and its treatment. This enables physicians and other health professionals to establish an individualized treatment plan and to provide customized information, support, and psychological counseling, which helps to optimize the provided care.

## 1. Introduction

Gestational diabetes mellitus (GDM) is a transient metabolic disorder, characterized by hyperglycemia that occurs and is first diagnosed during pregnancy, and is expected to resolve after childbirth [1]. The onset of GDM puts a heavy strain on the positive connotation of pregnancy and is accompanied by feelings of fear, anxiety, uncertainty, a sense of losing control, elevated levels of stress, a poorer perception of one’s health, reduced self-worth, and impaired QoL [2,3,4]. Pregnant women perceive GDM as a powerful stressor based on the disease’s adverse impacts on all areas of their lives, and the threat it poses on them and their children by means of potential complications and long-term consequences [5]. While the assessment of QoL is an undisputed and inherent part of the evaluation of almost all medium- and long-term medical treatments, a disease-specific QoL questionnaire tailored to women with GDM does not exist in German-speaking countries. In the past years, research on QoL in GDM has gained momentum, whereupon primarily generic and health-related but also diabetes-specific QoL questionnaires have been used for the assessment [6]. This comes at the cost that important aspects of QoL within GDM are not sufficiently represented. Yet, while generic and health-related QoL questionnaires are an option to compare QoL across different diseases, they are less sensitive to changes in specific health aspects related to a particular disease [7,8]. Diabetes-specific instruments disregard the fact that women suffering from GDM expect their condition as temporarily limited to their pregnancy, unlike the chronic nature of diabetes mellitus type one or type two [4]. Furthermore, diabetes-specific and general or health-related QoL questionnaires exclusively focus on the patients themselves and overlook the pregnant woman’s meaningful concerns regarding her fetus or newborn [9]. Although general questionnaires for pregnant women include concerns about the fetus and newborn in QoL measurement, they incorporate just as few GDM-specific aspects as general QoL or mental health questionnaires: the burdensome adaptation to the requirements of GDM, like the adjustment in lifestyle with dietary changes, the increase in regular movement, the blood sugar measurement, and, when indicated, the insulin treatment, may have significant adverse effects on QoL [10]. The usage of numerous different types of questionnaires for QoL assessment leads to inconsistent study findings on this topic, resulting in heterogeneous recommendations. Accordingly, it seems reasonable to establish a solid foundation for further research by developing and validating an appropriate patient-reported outcome measure for QoL assessments in GDM. Mokhlesi et al. have completed important work by introducing the Persian questionnaire GDMQ-36 for the assessment of disease-specific QoL in women with GDM, which has been proven to be reliable and valid for the Iranian population. To our knowledge, it is so far the only questionnaire worldwide tailored to the QoL of gestational diabetics that incorporates all the mentioned aspects due to the fact that it is exactly tailored to the needs as well as disease- and treatment-related challenges gestational diabetics are facing. It contains 36 questions and consists of five aspects: (1) concerns about high-risk pregnancy, (2) perceived constraints, (3) complications of GDM, (4) medication and treatment, and (5) support [10]. After gaining permission from the Teheran-based research team, we aimed to test if the German version of the questionnaire would perform similarly after a precise translation and cultural adaptation, enabling its use in German-speaking countries. Ultimately, our objective was to develop and validate the first German GDM-specific QoL questionnaire, encompassing all the aforementioned aspects of QoL that are meaningful from the pregnant women’s perspective but have been overlooked until now.

## 2. Relevance

The prevalence of GDM is continuously increasing in the context of the global epidemic of obesity and diabetes [11]. According to the International Diabetes Federation, 16.7% of pregnancies were affected by hyperglycemia in 2021, of which 80.3% were due to GDM [1]. In German outpatient care, the prevalence of GDM added up to 13.2% in 2014/15, increasing from 8% to 26% in women over 45 years old. [12]. These results align with our own observations at our tertiary care center, where 14.9% of pregnant women were affected by GDM in 2017. By 2020, this percentage had increased to 18.9%. To minimize the potential short- and long-term consequences of GDM, consistent metabolic control is mandatory, which requires the women’s willingness to take an active part in treatment. At this juncture, it is inevitable to acknowledge the women’s personal viewpoint on their condition, and to consider any impairments and problems related to their disease and its treatment, since GDM adversely influences their lifestyle as well as their psychological comfort and well-being [13,14]. Especially in lifestyle diseases such as diabetes and GDM, which require a high degree of self-management, a patient’s QoL has a significant impact on the success of therapy due to its link to adherence and clinical outcome [13,15]. Aspects of QoL are particularly important for the acceptance and thereby the implementation of diagnostic and therapeutic procedures in women with GDM (e.g., intensified insulin treatment—fear of hypoglycemia or fetal impairment), because they could reduce the women’s adherence via an adverse effect on QoL despite proven treatment efficacy. Additionally, it has been suggested that an improvement in care should occur on the basis of women’s personal living conditions and biopsychosocial status [13,15].

By means of the results of our study, we hope to promote the following subject areas:

(1) Improvement of quality and outcomes of GDM care
  By conducting frequent QoL measurements with our GDM-specific questionnaire, we can provide a comprehensive understanding of the pregnant women’s personal living conditions. In our prospect, this means that this questionnaire has to be filled in as promptly as possible after the pregnant women are diagnosed with GDM and subsequently approximately every two weeks, e.g., in line with their regular obstetrical appointments. Throughout this process, clinicians are able to identify the women’s expectations, special health care needs, treatment difficulties, and impairments in everyday life early on, allowing for appropriate interventions, even if the woman is not willing or able to talk about certain topics with her attending obstetrician. By this means, obstetricians will be able to choose personalized treatment options based on each woman’s unique living conditions and to offer tailored information, targeted support, and psychological counseling, which will optimize the care provided to each woman. The resulting improvement in QoL instills a sense of security and trust in the attending physician and treatment procedures, empowering the women to actively participate in the therapeutic process, ultimately leading to high levels of satisfaction with the GDM care received [6,7,11,13,14,16].

(2) Quantitative evaluation and improvement of diagnostic and therapeutic procedures
  Similar to other disease-specific questionnaires, our GDM-specific questionnaire will assess relevant health aspects and detect even minimal changes in a patient’s health status and health-related concerns in more detail and depth. This will enable a sensitive measurement of the pregnant women’s QoL, which holds significant practical importance for the comparison of diagnostic or therapeutic approaches in GDM care. For instance, our questionnaire could be used for cause analysis in the comparison of one therapeutic (or diagnostic) procedure against another, e.g., when both procedures are equal in their theoretical outcome, but one of them has been shown to be superior in a clinical setting via a much better adherence and overall outcome. Based on the assessment of the women’s health-related QoL with our questionnaire, it would be possible to show that the procedure that performs worse has a major negative impact on the women’s QoL, which is why the gestational diabetics discontinue their therapy and, therefore, have a worse outcome. Additionally, the questionnaire would be able to examine the proper reasons for the occurring differences in a clinical setting via a precise analysis of the women’s treatment-related concerns or problems with the new therapy, which lowers their QoL in such a way that they all continue their new therapy erratically or even discontinue it altogether. For instance, a pregnant woman could interrupt her newly administered insulin because of concerns that it might harm her fetus, because of reoccurring hypoglycemia symptoms, or because it is inconvenient for her to inject it at work, on the train, or in a restaurant. Hereby, copious interviews with probands and further test stages for causal research could be avoided [6,7,9,16]. This example shows that a solid and scientifically sound evaluation of the diagnostic and therapeutic procedures is of great importance, particularly in the context of health economics. Additionally, new technological advancements such as continuous glucose monitoring systems and insulin pumps, new medications, new lifestyle intervention strategies, or app-based monitoring methods, specifically designed for gestational diabetics, could be compared to the current standard GDM care with respect to their impacts on pregnant women’s health-related QoL by using our GDM-specific questionnaire. Thus, the most progressive diagnostic and therapeutic procedures could be chosen based on women’s QoL as one of the main metrics.

(3) Shift of focus regarding primary outcome objectives
  Traditionally, metabolic control and a favorable clinical outcome for a mother and her newborn were the primary therapy goals in GDM treatment. However, in recent years, a shift has occurred, emphasizing the need for a comprehensive assessment of women’s health and its appreciation as a whole. Instead of solely focusing on physical treatment goals, this shift highlights women’s perspectives on their conditions and the challenges they face in dealing with them [13,14]. For this reason, it was recommended to acknowledge the improvement of QoL as an equally important therapeutic goal as metabolic control and avoiding complications. Furthermore, it was suggested that future therapy optimization and adjustments should not only be based on physical factors but also on the individual circumstances of living and the patient’s biopsychosocial status [13]. Similarly, the WHO promotes a holistic health care approach whose primary goal is the patient’s well-being [16]. The same applies to the field of research: For many years, studies on GDM exclusively focused on the medical management and the risks for both mother and fetus, while only a few have explored how women with GDM experience their pregnancies and how the condition influences their QoL and perception of health [11,14]. Albeit QoL was increasingly measured in clinical research revolving around GDM over the years, these studies were not primarily intended to increase women’s QoL. Hence, their health-related QoL seemed to be more of a byproduct or a secondary outcome rather than the researchers’ primary focus of interest [7,14]. Given the higher vulnerability of women suffering from GDM, its continuously increasing prevalence, and the prospective provision of our GDM-specific questionnaire for QoL measurement, it would be appropriate to consider QoL as the main focus of intervention studies more frequently—recognizing its significance for expectant mothers and empowering them to enjoy their pregnancy with minimal stress under the given constraints.

## 3. Materials and Methods

### 3.1. Ethics

The planned study will respect the ethical standards of the Declaration of Helsinki and was given the approval of the local Ethics Committee (registration number A 2021-0142). After the pregnant women have been diagnosed with GDM by their outpatient gynecologist using a 75 g oral glucose tolerance test (oGTT) in accordance with the IADPSG criteria, they are regularly referred to an appointment at our outpatient care facility at the University Gynecological Hospital and Polyclinic of Rostock. They will be informed about the aims and the procedures of the study in oral and written form and will provide their written permission afterward. As no specific medical interventions will be conducted in our study, there is a relatively low risk of negative side effects on the female study participants. Conceivably, pregnant women may be confronted with unpleasant topics like possible short- and long-term complications of GDM while filling in our questionnaire, which could potentially cause feelings like fear, uncertainty, disappointment, anger, distress, or concerns about their own health and that of their unborn [2,3,5,13,14]. But these are all adverse psychological effects that could occur either way in the natural course of a pregnancy complicated by GDM and are rare occurrences while filling in a QoL questionnaire.

### 3.2. Aims of the Study

1. Translation of the Persian questionnaire GDMQ-36 [10] after gaining permission: forward and backward translation via native speaker and translation agency.
2. Expert ratings and pretests with cognitive debriefings and structured interviews with women suffering from GDM for the evaluation of comprehensibility, face and content validity.
3. Pilot testing and validation of the preliminary questionnaire to investigate the psychometric performance of the measures in terms of validity and reliability.

### 3.3. Design and Methods

We chose a mixed-methods approach for the study design, collecting qualitative as well as quantitative data. Firstly, we performed an expert survey with professionals as well as qualitative pre-testing using the cognitive debriefing method “think aloud” and structured interviews with patients. These qualitative methods are used to adopt the conceptual model, which forms the basis for our measurement model as well as to provide the content of the wording of items. Based upon this, this preliminary questionnaire will be initially piloted and finally validated using quantitative methods, aiming at testing various indicators of psychometric performance based on two larger independent samples. Those indicators feature different types of reliability and validity, described in more detail in Section 3.5.2. Quantitative methods that will be used for psychometric analysis include, amongst others, e.g., confirmatory factor analysis on the instrument level, reliability analysis on the scale level, and differential item functioning analysis on the item level.

### 3.4. Study Participants

#### 3.4.1. Sample Size

To ensure an increased generalizability of our results, we aim to represent the general GDM population as realistic as possible. For this reason, pregnant women with either dietary or insulin-treated GDM, coming from various sociodemographic backgrounds, will be included in the study.

Pretesting:

- For the expert ratings, we will include internal and external medical personnel, including gynecologists/obstetricians, endocrinologists, diabetes nurses, midwives, and dieticians. They will be contacted via email or orally. We aim for a total of 20 experts (*n* = 10 each, internal and external).
- Moreover, we will perform two pretests with women suffering from GDM. In the first cognitive debriefing, our focus will be on recruiting *n* = 30 patients, taking into account the common distribution of three main factors: normal weight vs. obesity, nullipara vs. multipara, and dietary vs. insulin treatment [17]. After a cultural and linguistic adaptation of the questionnaire, we will conduct another cognitive debriefing with patients until we accomplish a satiation of information assets. Roughly, we will try to obtain at least *n* = 10 patients.

Piloting and validation:

- There are still unknown factors such as the exact size of the item pool and item communalities as well as the number and eigenvalues of the factors to calculate the exact sample sizes needed for piloting and validating the instrument. However, we assume an estimated item pool of approximately 40 ± 10 items, and the approximate ratio of the number of cases to items is roughly 4:1 (ranging from 3:1 to 5:1). Thus, for the piloting and validation study, we need a sample size of *n =* 200 women with GDM, based on complex psychometric procedures like the planned confirmatory factor analysis. This number of cases seems to be sufficient for the analyses to be performed.

#### 3.4.2. Recruitment

Study participants will be recruited by the attending obstetricians of the gynecological outpatient department of the University Medicine Rostock. While women with GDM attend their first regular appointment at the outpatient department, the obstetricians will invite them to participate in the study in oral and written form if they meet the following inclusion criteria: the pregnant women must have a diagnosis of GDM within the current pregnancy, be at least 18 years old, have sufficient knowledge of the German language, and must not have any cognitive impairments. Women with a history of GDM being diagnosed once more in the current pregnancy will likewise be included, while their positive anamnesis regarding GDM is registered. Exclusion criteria include preexisting type I or type II diabetes mellitus, multiple pregnancies, as well as complications in their current pregnancy such as gestational hypertension, pre-eclampsia/eclampsia, or threatened preterm birth. The medical professionals for the expert ratings, who are required to have substantial work experience, will be recruited by the chief senior physician of the obstetrical department via email, internal mail, or in person.

#### 3.4.3. Study Assessment and Measures

Following an adaptation process consisting of an exclusion, addition, and modification of the previous items of the translated Persian GDMQ-36, the resulting preliminary version of our QoL questionnaire will be piloted and validated. In order to do so, study participants will also need to complete the following standardized and established QoL questionnaires during both the pilot and the validation phase: the WHOQOL-BREF as a generic questionnaire, the EQ-5D-5L and VR-12 as health-related, and the PAID-20 as a diabetes-specific questionnaire for the QoL measurement. Additionally, the pregnant women’s socio-demographic data and medical histories will be collected. For test-retest reliability, 50 women will have to complete our GDM-specific questionnaire again after two weeks during the piloting and the validation phase (Table 1).

The “World Health Organization Quality of Life Bref”, in short WHOQOL-BREF, is a short version of the WHOQOL-100 questionnaire, containing 26 instead of 100 questions. Measuring the general QoL in healthy and sick individuals, its questions refer to the person’s experienced situations of the last two weeks. The first two questions revolve around one’s general QoL and the general perception of one’s health. The following 24 questions about various aspects of QoL are arranged in four domains: physical, psychological, social, and environmental. Patients rate their QoL on a five-point Likert scale with varying response options for the posed questions (e.g., response options of item no. 1: 1 = “very bad”, 2 = “bad”, 3 = “moderate”, 4 = “good”, and 5 = “very good”; response options of item no. 8: 1 = “not at all”, 2 = “a bit”, 3 = “moderate”, 4 = “fairly”, and 5 = “very much”). The total score can reach from 0 to 100, whereas higher scores indicate a higher QoL, with 100 representing the best QoL possible. Cronbach’s Alpha values have been shown to be between 0.76 and 0.88 in the German version of the WHOQOL-BREF [18].

The “European Quality of Life 5 Dimensions 5 Level Version”, in short EQ-5D-5L, assesses the current health-related QoL independently of preexisting diseases. It covers the five domains of mobility, self-supply, daily activities, pain/physical discomfort, and anxiety/depressiveness. Choosing one out of the five response options, respectively, (1 = “no problems”, 2 = “mild problems”, 3 = “moderate problems”, 4 = “big problems”, and 5 = “not able”), the patient allows a precise evaluation of each domain and, therefore, their current health, whereby lower scores represent a better health status. Moreover, the patients have to rank their current health on a visual analog scale (VAS) reaching from 0 to 100, with 0 being the worst and 100 being the best health status imaginable for the patient [19].

The ”Veterans RAND 12 Item Health Survey”, in short VR-12, is a derivative of the VR-36, which is similar to the frequently used SF-36 and will be applied to evaluate the subjective health status of pregnant women. It consists of questions about general health, physical functioning, physical pain, physical role-functioning, mental health, social functioning, as well as mental role functioning and vitality, which are all covered with from 1 to 3 questions each, totaling 12 questions. Responses vary between 3 and 6 options and different appellations (e.g., response options of item no. 2: 1 = “yes, strongly restricted”, 2 = “yes, a little restricted”, and 3 = “no, not restricted at all”; response options of item no. 10: 1 = “always”, 2 = “usually”, 3 = “quite often”, 4 = “sometimes”, 5 = “seldom”, and 6 = “never”). The patient’s general health status can be evaluated independently of preexisting diseases in two main scores: the “Physical Component Summary” (PCS) and the “Mental Component Summary” (MCS), which are both standardized and allow for comparisons [20]. 

The unidimensional “Problem Areas in Diabetes”, in short PAID-20, had been designed for type I and II diabetics and can be used independently of their type of treatment such as dietary measures, oral antidiabetics, or insulin injections. It is being applied to identify current emotional burdens in regard to various aspects of diabetes as well as depressive moods. The patient has to respond to 20 questions by choosing an answer from a five-point Likert scale reaching from 0 (“no problem”) to 4 (“serious problem”). Addressed topics revolve around hypoglycemia, medical complications, nutrition, restrictions, support, acceptance of illness, as well as feelings of anxiety, worries, guilt, discouragement, overburdening, and exhaustion. The higher the patient scores, the more severe the patient’s emotional, diabetes-related problems, so the total score allows a rating of the extent of their diabetes-related burdens. The German PAID-20 has shown a highly satisfying reliability (Cronbach’s Alpha = 0.93; test-retest reliability = 0.75) and proven construct validity and concurrent validity [21].

### 3.5. Data Evaluation

#### 3.5.1. Data Collection and Management

The handling of data related to and resulting from our study, including obtained patient consent, data collection, transfer, analysis, and storage of data, will comply with current data protection regulations. Our questionnaires and the forms for socio-demographic and medical data will be copied by the study nurse and handed out by the obstetrician to each eligible woman. After completing everything on-site, the women return all documents to the nurses. The second GDM-specific questionnaire for the retest will be returned at the next appointment in the outpatient department of the University Medicine Rostock, which is about 2–4 weeks after the first one. The questionnaires and forms will be filed away by the study nurse, and entered into a database form by a scientific assistant. Afterward, the original questionnaires and forms will be stored in locked cabinets according to the data protection regulations. 

#### 3.5.2. Data Analysis

After an initial piloting phase to examine the descriptive parameters (e.g., response choice frequencies and patterns, location and dispersion parameters, floor and ceiling effects, missing data, and outliers) and psychometric performance on the item, scale, and instrument levels based on the classical test theory and item response theory, and adjusting our questionnaire accordingly, we will validate its final version. This will involve determining its psychometric quality through confirmatory factor analysis and assessing the psychometric characteristics of the scales, especially reliability (internal consistency, split-half-reliability, test-retest reliability) and validity (convergent and divergent validity, known-groups validity), and the items using both classic and modern test theories (e.g., detecting potential item bias by differential item functioning analysis). Retests on a subpopulation of participants (*n* > 50) will allow for statements about the test-retest reliability and sensitivity to change. Simultaneously, established questionnaires for assessing global (WHOQOL-BREF), health-related (EQ-5D-5L, VR-12), and diabetes-specific QoL (PAID-20) will also be administered, enabling a comparative analysis of convergent and divergent validity. Known-group validity will be investigated by testing hypotheses for known differences in QOL between various groups using GDM-QOL scores.

## 4. Discussion

Our study is expected to create the following outcomes:

(1) Patient-driven re-conceptualization:
  In cognitive debriefings and structured interviews, the pregnant women could potentially reveal a couple of relevant subjects that concern them and thereby influence their QoL adversely, which may not have been covered in other instruments. Otherwise, it is conceivable that in the end some aspects, which one currently attributes great impact to, could be less important for women than previously thought. If this presumption comes true, our GDM-specific questionnaire will be able to capture the very aspects that are actually influencing the QoL of pregnant women with GDM.

(2) Disease-specific assessment:
  Through the questionnaire, the women’s individual expectations, needs, impairments, problems, and experiences regarding different treatment options or struggles with lifestyle changes (e.g., healthier nutrition, increased exercise) can be assessed more sensitively than with other QoL questionnaires. Through the application of the GDM-specific instrument, women’s overall QoL or the impact of a particular intervention on it can be assessed more accurately due to the sensitivity of this disease-specific measure [8,10].

(3) Tailored health care:
  Especially in lifestyle diseases such as GDM, there is a necessity to assess each woman’s needs individually: On the one hand, there are aspects that women with GDM are worried about or feel insecure about, such as implementing blood sugar measurements, insulin therapy, or managing the consumption of certain foods. As a result, they may need assistance and support in these areas. On the other hand, there are aspects they can handle very well on their own without further need for support. By facilitating a broader perspective of the pregnant woman’s personal viewpoint and attitudes, our GDM-specific questionnaire enables physicians to establish an individualized treatment plan and to allocate customized information, targeted support, and psychological counseling, which help to optimize and tailor the provided care to the personal needs and individual expectations of each pregnant woman [11,13]. Furthermore, these selective measures facilitate an efficient use of human resources in times of a shortage of physicians and other medical personnel.

(4) Care-relevant evaluation:
  Finally, this instrument allows for the assessment of the impact of certain procedures and interventions on women’s QoL, enabling precise comparisons of treatment options, targeted interventions, and the development of new therapies to become possible [7,10,16]. In addition, inconsistent study findings regarding QoL in GDM could be re-evaluated, which would make studies on this topic more comparable as well as more valid and reliable [10].

## 5. Limitations of the Study

This study will be conducted as a single-center prospective study. Thus, the included study participants may not be completely representative of the population of women with GDM in Germany or even other German-speaking countries like Austria and Switzerland. Hence, further validation may be needed. It could also be conceivable that women refusing to participate in the study belong to a certain social, ethnic, or other vulnerable group that will be underrepresented in the findings of our study due to their avoidance of participation.

## 6. Conclusions

The inclusion of relevant aspects of QoL is essential for evaluating new diagnostic and therapeutic procedures for numerous diseases, as well as for optimizing adherence among patients and subsequently improving clinical outcomes [11,13,16]. Thus, an assessment of QoL plays an increasingly inherent role in clinical studies [6,13]. Considering the huge societal impact of GDM, the availability of a validated GDM-specific questionnaire for German-speaking countries could lead to a broad utilization in multitudinous studies and, through this, contribute to an improvement of quality in GDM care. Against the background of the implementation of new technological developments in GDM care such as the use of systems for continual glucose monitoring, insulin pumps, the establishment of lifestyle interventions, or app-based monitoring methods, the utilization of our instrument will be able to facilitate a fundamental amelioration in a quantitative evaluation of the efficiency of these procedures, which at least is not of the utmost importance for health economics.

## Figures and Tables

**Table 1:** Questionnaires and information collected at varying times of assessment.

Study Assessments and Measures	Number of Items	Study Time Points			
		Pilot Study	Retest I	Validation Study	Retest II
General information					
Sociodemographic data	14	x		x	
Medical history	14	x		x	
Psychological instruments					
Item pool of the preliminary GDM-specific questionnaire	69	x	x	x	x
WHOQOL-BREF	26	x		x	
EQ-5D-5L	6	x		x	
VR-12	12	x		x	
PAID-20	20	x		x	

x = data collected at this point of time.

## Data Availability

The datasets used and/or analyzed during the current study are available from the corresponding author upon reasonable request.

## Abbreviations

QoL	Quality of Life
GDM	Gestational Diabetes Mellitus
GDMQ-36	Gestational Diabetes Mellitus Questionnaire 36
